# Caution Needed When Predicting Species Threat Status for Conservation Prioritization on a Global Scale

**DOI:** 10.3389/fpls.2020.00520

**Published:** 2020-04-28

**Authors:** Barnaby E. Walker, Tarciso C. C. Leão, Steven P. Bachman, Friederike C. Bolam, Eimear Nic Lughadha

**Affiliations:** ^1^Royal Botanic Gardens, Kew, Richmond, United Kingdom; ^2^School of Natural and Environmental Sciences, Newcastle University, Newcastle upon Tyne, United Kingdom

**Keywords:** conservation, machine learning, automation, IUCN Red List, extinction risk, transparency

## Introduction

The recent Intergovernmental Science-Policy Platform on Biodiversity and Ecosystem Services (IPBES) report highlighted the large scale of extinction risks to biodiversity (Díaz et al., 2019). Assessing species' extinction risk is vital for setting conservation priorities and the first step toward protecting particular areas or groups. A widely accepted approach to assess extinction risk, and a key source of data underpinning the IPBES report, is the IUCN Red List of Threatened Species (hereafter Red List). However, with only 9% of plants represented by assessments at the latest update (IUCN, 2019), slow progress in increasing Red List coverage of mega-diverse groups like plants has limited their inclusion in analyses of global conservation priorities (Venter et al., 2014; Betts et al., 2017; Di Marco et al., 2018). Responding to this problem, there is growing interest in speeding up the assessment process. Automation, particularly through machine learning, offers an attractive solution. However, we advocate caution before adopting it to help set global conservation priorities.

We draw on two recent examples from the literature (Pelletier et al., 2018; Stévart et al., 2019) that deserve attention as the largest studies to date that use machine learning or automation to predict the conservation status of plants. Each study recommends a protocol for rapidly generating preliminary conservation assessments, and both share the goal of using their preliminary assessments to highlight global or continent-wide conservation priorities. The potential impact of these studies merits careful scrutiny. Herein we highlight aspects of their design and reporting that can be improved so that future studies of this kind can have maximum impact.

## Large-Scale Approaches to Predicting Extinction Risk of Plants

In the most ambitious plant extinction risk prediction study to date, Pelletier et al. (2018) aimed to use machine learning to predict the conservation status of all known land plants. Using GBIF occurrences, Pelletier et al. trained separate random forest models for plants endemic to each continent, and a further model for plants native to more than one continent, to make predictions for all species with at least five occurrence records. Their best-performing set of models predicted extinction risk of over 150,000 species of land plants, with 73–82% of species predicted correctly as threatened or not threatened.

In a contrasting approach, Stévart et al. (2019) automated the calculation of certain metrics used in Criteria A and B for Red List assessments and evaluated species using a new approach: Preliminary Automated Conservation Assessments (PACA). Designed to be complementary but separate to the Red List schema, PACA comprises two systems. The first recognizes three main levels: Likely Threatened (LT); Potentially Threatened (PT) and Potentially Not Threatened (PNT). The PNT level is divided further into three sublevels: Likely Rare (LR), Potentially Rare (PR), and Likely Not Threatened (LNT). The second system recombines these sublevels with the first two main levels as follows: Likely Threatened & Likely Rare (LT+LR), Potentially Threatened & Potentially Rare (PT+PR), and Likely Not Threatened (LNT). The three levels of each system are intended as triage toward three subsets of Red List categories; Critically Endangered & Endangered, Vulnerable, and Near Threatened & Least Concern respectively. Stévart et al. use their first system to report that 31.7% of the tropical African flora is potentially threatened (i.e., assigned to LT or PT) and their second system to report their best performance metrics when comparing PACA predictions to published Red List assessments.

## Clarity in Reporting Performance

Clear and consistent reporting of method performance is paramount to enable readers to judge the reliability/credibility of published predictions. Both studies compare predictions to published IUCN Red List assessments to estimate method performance, as do previous studies (Bland et al., 2015; Darrah et al., 2017; Nic Lughadha et al., 2018). Accuracy, or its inverse the error rate, are most often quoted, but these give an incomplete picture of predictive performance. For predictions of extinction risk in particular, the number of not threatened species usually far outweighs the number of threatened species. It is therefore important to provide separate measures of how well a method correctly predicts species as threatened and not threatened. Two popular measures for these are sensitivity and specificity (Bland et al., 2015; Darrah et al., 2017; Di Marco et al., 2018), respectively.

Pelletier et al. report their random forest models to achieve predictive accuracies comparable to previous studies. However, neither sensitivity nor specificity are reported for any of their models. Without these measures readers cannot judge whether predicted numbers of threatened species are inflated by mis-predictions of not threatened species or depressed by mis-predictions of threatened species.

More concerning, however, is that Pelletier et al. use different classification thresholds when reporting model performance of predicted numbers of potentially threatened species. To evaluate the performance of their models, by default, they classify as potentially threatened species having predicted probability of being threatened exceeding 0.5. Then, citing caution as their motive, they use two higher thresholds (0.6, 0.8) to predict numbers of threatened species. They fail to report model performance at these higher thresholds, or how performance changes with the threshold, leaving readers unable to fully evaluate their estimates.

Pelletier et al. are not alone in highlighting model performance for one procedure and reporting predictions from another, losing a clear link between predictions and performance. As described above, Stévart et al. introduce two different systems within their PACA procedure. They use their first system (LT>PT>PNT[= LR+PR+LNT]) to generate their headline figure: a third of tropical African flora potentially threatened. But they use their second system (LT+LR > PT+PR > LNT) when reporting their best measure of performance—a sensitivity of 0.84 for the LT+LR level. They claim that PACA has high sensitivity, but in fact sensitivity for the LT level of system 1, used for their headline, is just 0.20. In contrast, had they used the better performing system 2, their headline figure would have been 70.1% of tropical African plants potentially threatened (compared to 31.7% reported). We commend Stévart et al. for providing the data needed to calculate this, however, their failure to establish a clear link between prediction and model performance renders their headline claim potentially misleading.

## Exploring the Effects of Modeling Choices

Large-scale automation of preliminary extinction risk assessments inevitably involves simplifications and choices in model building. Where authors fail to explore how such choices and simplifications may affect results, the value of their research is reduced as readers cannot know which choices are defensible.

Stévart et al. attempt to automate application of Criteria A and B for Red List assessments, but do not fully explore the effect of simplifications they make when calculating the required metrics. These metrics comprise, for each species, the area of occupancy (AOO), extent of occurrence (EOO), number of threat-defined locations, number of occurrence records in declining habitats, and expected reduction in AOO. They calculate EOO and AOO in a standard way but their novel methods for estimating the number of threat-defined locations and decline in habitat introduce simplifications inconsistent with IUCN recommendations. The reader cannot tell if these simplifications are reasonable in the absence of any exploration of their impact on the performance of the PACA method.

**Figure 1:**
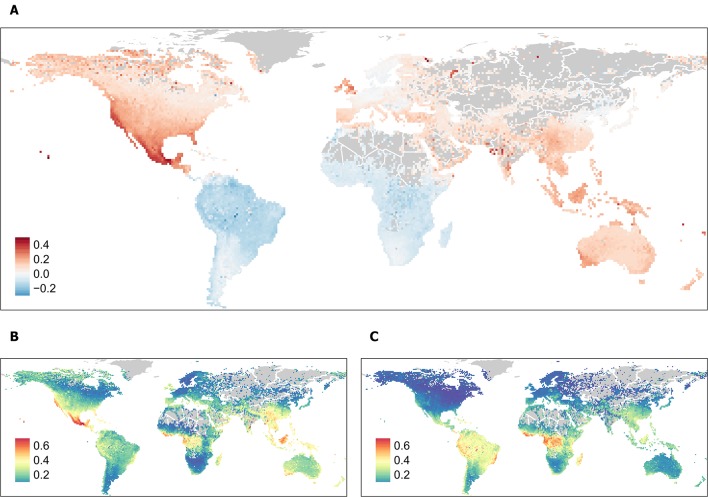
**(A)** Difference in average per grid-cell probability of being threatened between **(B)** our reproduction of the continental predictions in Pelletier et al. and **(C)** predictions made from a single global model following the same protocol and using the same species. Map **(A)** highlights regions with potentially over-stated extinction risk (red), and potentially under-stated extinction risk (blue).

Pelletier et al. choose to build separate random forest models for each continent but do not calibrate the predicted probabilities of threat, making these predictions incomparable between continents. This reduces the reliability of probability maps for identification of global conservation priorities, a goal of the paper. We followed the protocol of Pelletier et al. as closely as possible, using the data they provided, to: (i) reproduce their map of continental predictions and (ii) produce a map of predictions from a single global model (Figure 1). Comparison suggests that predicted threat levels were overstated for North America and understated for South America and Africa. In our view, their choice to build separate models for each continent, combined with their data-cleaning process and down-sampling scheme, introduced these discrepancies into their predictions.

## Treatment of Threat

Documentation of threats faced by species is central to Red List assessment but challenging to automate due to the specificity of IUCN concepts, such as threat-defined locations, combined with the diversity of threats and variability in how they may affect different species at the same location. Stévart et al. attempt to document threats in the calculation of two of their metrics described above. First they identify which occurrences face habitat decline and loss, using data on land cover and mining concessions. This approach omits some species-specific threats but incorporates some information about plausible threats. Second they estimate the number of “threat-defined locations” for each species but their estimate is based almost entirely on the spatial distribution of species occurrences, incorporating no information about threat. Stévart et al. make clear that both approaches are simplifications. However, the links made between their approaches and the IUCN Red List criteria risk leading readers to think that these approaches are more consistent with IUCN guidelines than they actually are. Furthermore, while increasing availability of threat data may improve these approaches, performance may still be limited by the way in which threat data are used. Stévart et al.'s approach to estimating the number of threat-defined locations in particular has the potential to inflate numbers of species identified as threatened by being overly conservative (Nic Lughadha et al., 2018).

In contrast, Pelletier et al. make no attempt to directly incorporate threat in their models. Their best-performing models includes only environmental and geographical predictors, whereas previous studies show human impact predictors as among the most important in modeling extinction risk (Darrah et al., 2017; Di Marco et al., 2018). We consider it important to incorporate measures of threat as predictors in such models, as well as others based on information like habitat type that may be a proxy for extinction risk. These predictors, as well as possibly improving the quality of predictions, would also enhance the utility of results concerning the relative importance of predictors.

## Limitations of Biodiversity Data

Automated methods and machine learning models for generating preliminary conservation assessments are necessarily based on widely available biodiversity data. These data, including plant occurrence records, have well-documented biases and gaps (Meyer et al., 2016). Furthermore, plant species assessed for the IUCN Red List are not randomly selected but reflect taxonomic and geographic preferences and the imperative to list threatened species. Understanding and addressing these biases is important if automated preliminary assessments are to be reliable/useful.

Pelletier et al. take steps to account for the imbalance of threatened and not threatened species in the IUCN Red List by down-sampling when building their models. They also attempt to address geographic biases in species assessed by using separate models for each continent.

However, their process for data cleaning may introduce further biases. Pelletier et al. remove all species with 4 or fewer georeferenced occurrences, reducing representation to <50% of known species (c. 150,000). Species with such limited numbers of occurrence records are very numerous and tend to be rare (Enquist et al., 2019), so this pruning inevitably resulted in removal of disproportionate numbers of threatened species.

This is a familiar problem in conservation science (Duffy et al., 2009): in addressing urgent needs to understand extinction risk and prioritize actions we must avoid focusing primarily on the fraction of plant species that are sufficiently well-documented to model, potentially overlooking under-collected areas and those richest in range-restricted species likely to be threatened. One potential way forward is to use coarser-scale but more complete distribution data, which has proved useful in predicting conservation status of bulbous monocot plants (Darrah et al., 2017).

## Toward Better Predictions

Machine learning and automation are undoubtedly useful for accelerating assessment of plant extinction risk, as already demonstrated in smaller-scale studies of taxonomically- (Bland et al., 2015; Darrah et al., 2017) or geographically-defined groups (Leão et al., 2014; Nic Lughadha et al., 2018). However, as their use becomes more widespread and is advocated to inform global conservation priorities (Wearn et al., 2019), we must demand rigor and transparency in their application and scrutinize their predictions thoroughly.

We seek to ensure that future predictions factor in lessons learned from previous studies and make dedicated efforts toward reporting method performance clearly, exploring the effects of modeling choices, including sensible treatment of threat and accounting for limitations of the underlying plant data. Such efforts should help to generate increasingly trustworthy predictions as input for conservation decision-making.

## Author Contributions

BW, TL, SP, FB, and EN all conceived of the article, contributed ideas, and contributed to the writing. BW prepared the first draft and made the figure.

## Conflict of Interest

The authors declare that the research was conducted in the absence of any commercial or financial relationships that could be construed as a potential conflict of interest.
